# Robustness of Significant Dichotomous Outcomes in Randomized Controlled Trials in the Treatment of Patients with COVID-19: A Systematic Analysis

**DOI:** 10.1007/s44231-022-00027-y

**Published:** 2023-01-12

**Authors:** Qi Liu, Hong Chen, Yonghua Gao, Changju Zhu

**Affiliations:** 1 Emergency Department, The First Affiliated Hospital of Zhengzhou University, Zhengzhou University, No. 1st, Jianshe Eastern Road, Zhengzhou, Henan Province People’s Republic of China; 2 Department of Translational Medicine Center, The First Affiliated Hospital of Zhengzhou University, Zhengzhou University, Zhengzhou, Henan Province People’s Republic of China; 3 Department of Respiratory and Critical Care Medicine, Shanghai Pulmonary Hospital, Tongji University School of Medicine, Shanghai, People’s Republic of China; 4 Henan Medical Key Laboratory of Emergency and Trauma Research, The First Affiliated Hospital of Zhengzhou University, Zhengzhou University, Zhengzhou, Henan Province People’s Republic of China

**Keywords:** Fragility index, Fragility quotient, Coronavirus disease 2019 (COVID-19), Randomized controlled trials, Robustness

## Abstract

**Purpose:**

Significant results of randomized controlled trials (RCTs) should be properly weighed. This study adopted fragility index (FI) to evaluate the robustness of significant dichotomous outcomes from RCTs on coronavirus disease 2019 (COVID-19) treatment.

**Materials and methods:**

ClinicalTrials.gov and PubMed were searched from inception to July 31, 2021. FIs were calculated and their distribution was depicted. FI’s categorical influential factors were analyzed. Spearman correlation coefficient (*r*_s_) was reported for the relationship between FI and the continuous characteristics of RCTs.

**Results:**

Fifty RCTs with 120 outcomes in 7869 patients were included. The FI distribution was abnormal with median 3 (interquartile range 1–7, P = 0.0001). The FIs and robustness were affected by the outcomes of interest, various patient populations, and interventions (T = 18.215,16.667, 23.107; P = 0.02,0.0001, 0.001, respectively). A cubic relationship between the FIs and absolute difference of events between groups with R square of 0.848 (T = 215.828, P = 0.0001, R square = 0.865) was observed. A strong negative logarithmic relationship existed between FI and the P value with R square = – 0.834.

**Conclusion:**

The robustness of significant dichotomous outcomes of COVID-19 treatments was fragile and affected by the outcomes of interest, patients, interventions, P value, and absolute difference of events between the groups. FI was an useful quantitative metric for the binary significant outcomes on COVID-19 treatments.

**Registration:**

PROSPERO (CRD42021272455).

**Supplementary Information:**

The online version contains supplementary material available at 10.1007/s44231-022-00027-y.

## Introduction

Since the outbreak of the coronavirus disease 2019 (COVID-19), the pandemic has spread worldwide. Owing to the high infectivity [1], paucity of effective therapies [2], high hospitalization, and fatality rate [3], it poses a great threat to human life and challenges the health care systems. A large amount of research has come forth to overcome this global threat [4]. Great progress has been made in virology, diagnosis, prevention, and treatment [5]; some of them have been elucidated by randomized controlled trials (RCTs) [6]. Commonly, evidences from RCTs were considered to be vital in the evidence-based medicine pyramid [7]. The threshold, P-value of < 0.05, is the most commonly adopted criterion to judge the statistical significance. However, a “significant” result is not equal to a true treatment effect since it is also affected by the sample size, the number of events and participants lost to follow-up [8, 9]. In other words, P-value metric itself is worthy of concern [10]. In addition, data validation and trial integrity were affected by the elusory coronavirus to a certain extent [11, 12]. Therefore, potential bias and even misleading results of RCTs should be especially concerning in this unique era [13, 14]. Although effective therapeutic options and guidance are urgently required [15], proper interpretation of the new findings is crucial, especially to identify the fragile conclusions that could easily be invalidated by upcoming trials and thereby avoid excessive confidence in the significant results of RCTs [2, 12].

Therefore, a method to measure the robustness of the results of RCTs and assist the clinicians’ proper interpretation of the findings could be useful. In recent years, the fragility index (FI) has been considered as a meaningful metric [SPS:refid::bib16]^16^. For a statistically significant dichotomous outcome, FI is equal to the number of participants that need to be shifted from the nonevents to events aiming to change the statistically significant difference to a nonsignificant difference when reanalyzed by the Fisher’s exact test [17]; the higher the FI, the robuster the result, and vice versa. This tool has been recommended in critical care medicine [18, 19], anesthesiology [20], trauma, and surgical remedy [17]. Thus, we adopted FI to evaluate the robustness and determine the influential factors of FI, which would aid the clinician in weighing the current findings of the RCTs.

## Methods

### Overall Design

The protocol of this systematic analysis was approved by the First Affiliated Hospital of Zhengzhou University and registered in PROSPERO (CRD42021272455). All methods were performed in accordance with the relevant guidelines and regulations including the commonly used PRISMA guideline [21]. Ethical review and informed consent were waived for this type of study by the Ethics Committee of the First Affiliated Hospital of Zhengzhou University.

### Search Strategy and Eligible Criteria

To identify the relevant RCTs, ClinicalTrials.gov and PubMed databases were searched from inception to July 31, 2021. The keywords used were COVID, COVID-19, and severe acute respiratory syndrome coronavirus 2 (SARS-CoV-2). In PubMed, the preliminary searches were filtered using the RCT filter; in ClinicalTrials.gov, the aforementioned keywords were searched first without restrictions and then filtered further by “interventional clinical trial” and “with results.” In the end, the filtered search results were judged further according to the abstract and/or full texts manually. The inclusion criteria for RCTs were as follows: (1) participants were confirmed SARS-CoV-2 infected patients, (2) RCT with parallel groups of 2 or 2 × 2 factor design, (3) 1:1 allocation to intervention group and control group, and (4) at least one outcome was categorical data and the difference between the groups was statistically significant. The exclusion criteria were as follows: (1) designed as cluster RCTs, cross RCTs, and RCTs with more than 2 parallel groups, (2) sample allocation was not 1:1, (3) both the primary and secondary outcomes were not reported as dichotomic variable or were presented as time-to-event binary data, (4) the difference of the binary result was not statistically significant, and (5) systematic reviews, meta-analyses, descriptive studies, analytical studies, diagnostic tests, theoretical studies, observational studies, and RCTs with non-human subjects. The eligibility of the searched studies was assessed by two investigators (Q.L. and H.C.). Divergent opinion was solved through discussion until a consensus was reached, otherwise, the third investigator (Y.G.) would make a final conclusion.

### Data Collection

Two researchers (Q.L. and H.C.) extracted the data independently using a standardized form and the data were collected in one final form. For each eligible RCT, we extracted the data of the significant dichotomous outcomes, including the number of events, non-events for each parallel group, and the corresponding P-value. We recorded the boundary value, 0.001, as the P value if it was reported as < 0.001 to make it computable. We also extracted the following characteristics: journal name, year of publication, study design (method of assignment, blinding or not), and number of participants who were lost to follow-up. Disputed data were validated and determined by the third investigator (Y.G.).

### Risk of Bias Assessment

The Jadad scale [22], a tool with the best validity and reliability for assessing the methodological quality of RCTs [23], was used to appraise the possible risk of bias. This scale included three parts: randomization (0 score, quasi RCT; 1 score, studies in which randomization was stated without describing how the random number was generated; and 2 score, RCTs that reported the correct random number generation method), double blinding (0, whether blindness had not been mentioned; 1, blindness had been mentioned but had not stated how to maintain the efficiency of the blindness; and 2, trial that adopted sound method, such as placebo to ensure a blinded trial), and withdrawals and dropouts (0 score, studies in which dropouts had not been described although the number of analyzed patients was less than the number of recruited patients, 1 score, studies in which the number and the reasons of dropouts had been stated).The RCTs with a score ≤ 2 were considered low quality and a score ≥ 3 high quality.

### FI and FQ Calculations

In principle, the index, FI, was calculated by removing a nonevent to the group with smaller number of events until the two-sided P-value was ≥ 0.05 by Fisher’s exact test. FI was considered as zero if the significant difference vanished after just being reanalyzed by Fisher’s exact test. In actual calculation, we resorted to an online FI calculator [24]. Fragility quotient (FQ) was computed by the ratio of the FI score to the total sample size of the corresponding trial.

### Statistical Analysis

Continuous outcomes with abnormal distribution tested by Kolmogorov–Smirnov were reported as median and interquartile range (IQR). For discontinuous outcomes, the data are expressed as the number of a certain event and a percentage. The comparison between/among the subgoups was analyzed with the Mann–Whitney *U* test or Independent-Samples Kruskal–Wallis Test according to the number of subgroups divided by certain characteristics. Spearman correlation coefficient was calculated and curve estimation was performed to determine the relationship between the FI and characteristic reported as continuous data. P value < 0.05 was considered as a statistically significant difference. The employed statistical software included SPSS version 26.0 (IBM Corp., Armonk, NY) and Stata 16 (Stata Corp., College Station, TX, USA); OriginalPro 8 (OriginLab, Northampton, USA) was adopted to plot figures.

## Results

### Literature Search and Identification of the Studies

**Fig. 1:**
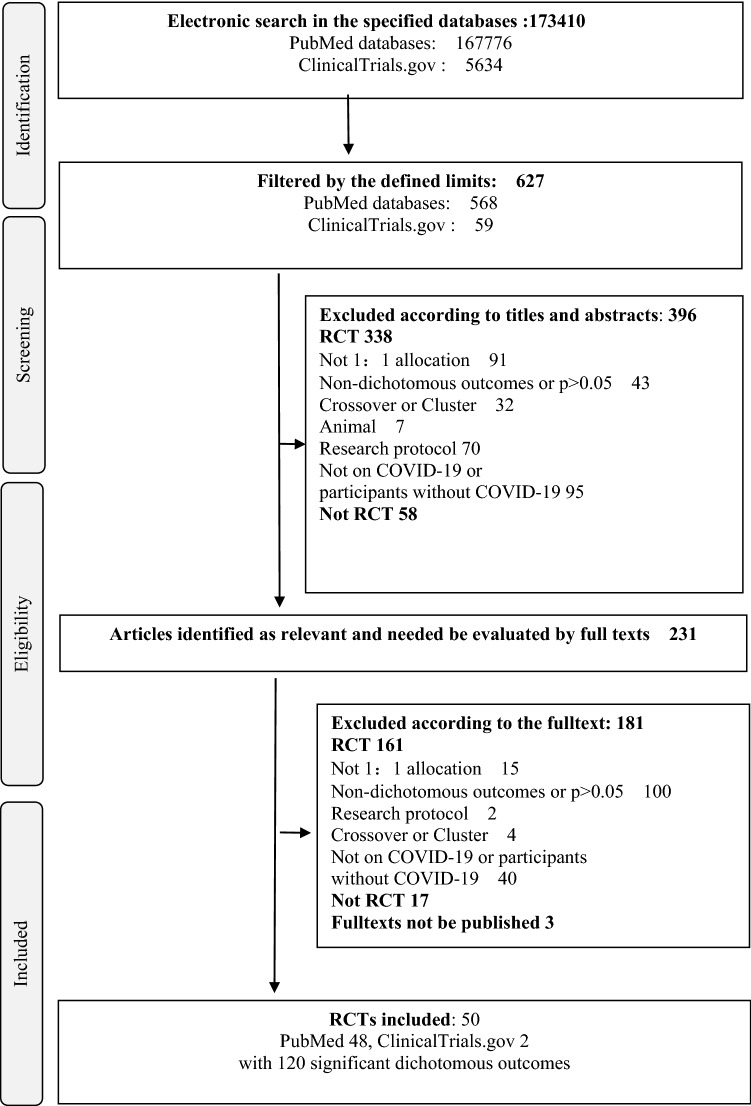
Flow chart of the trial inclusion in this study. RCT, randomized controlled trial; COVID-19, Corona Virus Disease 2019

Overall, we found 173,410 (PubMed databases, 167,776; ClinicalTrials.gov 5634) recordings according to the search strategy; 627 articles were found after being filtered by the previous defined limits. A total of 231 reports were identified as relevant ones judging from the titles and abstracts; further evaluation was performed based on the full texts and the eligible criteria. Finally, 50 RCTs with 120 significant dichotomous outcomes were considered valid and were included in the subsequent analysis (Fig. 1).

### Characteristics and Quality Assessment of the Included RCTs

In this study, we included 21 multi center and 29 single center RCTs covering 7869 patients with COVID-19 with different levels of severity. The RCTs were performed in Iran, Brazil, United Kingdom, United States, China, India, Canada, Italy, and the other countries, additionally, two RCTs were completed in multiple countries (Supplementary Table S2. References of the included studies). The interventional strategies included immunomodulatory agents, convalescent plasma therapy, glucocorticoid, antiviral drugs, respiratory support method, local traditional medicine, anticoagulation, inflammation inhibitors, and others. There were 17 (34%) RCTs in which placebo was adopted as the control strategy and 25 (49.02%) RCTs in which only standard treatments according to the guidelines of the time were applied in the control group. The included studies reported 41 primary and 79 secondary dichotomous outcomes with significant difference between the intervention and the control groups. Forty-two RCTs (84%) were rated as ≥ 3 JADAD score and 22 (44%) had depicted proper randomization concealment arrangements. The median total sample size was 69 with an IQR of 52.25–134.75, of which, 35.5 (IQR 26.5–67.75) were in the intervention group and 34.5 (IQR 25.75–66) were in the control group; the median total event was 32 (IQR 16.25–52.5) and 11 (IQR 3–25.75) in the intervention group and 17.5 (9–34.75) in the control group. The median total dropout was 0 (IQR 0–6) with a maximum of 56. More characteristics are reported in supplementary Table S1, which are further summarized in supplementary Table S3.

### The Pooled Results and Corresponding FIs and FQs of the Reported Significant Outcomes

As shown in Table 1, the pooled results indicated that intervention strategies significantly reduced the adverse events, clinical deterioration rate, need for hospitalization or intensive care unit (ICU), need for positive pressure breathing support, severe malfunction of key organs, and increased the clinical improvement rate, viral nucleic acid negative rate, and symptom control rate (P < 0.05); however, they did not decrease the mortality (odds ratio [OR] 0.55 [0.26, 1.18], P = 0.124). FIs varied in the different outcomes of interest, the robustness of reducing adverse events (FI: median, 6.5; IQR, 2–21.25) was the strongest and the robustness of effect on mortality, hospitalization, and severe malfunction of key organs was very weak (FI, median 1). The overall difference was significant (T = 18.215, P = 0.02). The subsequent pairwise comparisons implied that the FIs of the outcomes concerning the adverse events (median 6.5, IQR 2–21.25) were larger than those of admission to the hospital or ICU (median 1, IQR 0–1.5, T = 50.321, P = 0.017), clinical improvement rate (median 2, IQR 0.5–9, T = 23.887, P = 0.02), clinical deterioration rate (median 2, IQR 1–3.75, T = 30.02, P = 0.007), mortality (median 1, IQR 0.5–4.5, T = 31.723, P = 0.015), and aggravated malfunction of key organs (median, 1; IQR, 0.25–3.75; T = 34.779; P = 0.004);

**Table 1:** The pooled results of reported dichotomous outcomes with significant difference

	Studies/outcomes	Event/sample in intervention group	Event/sample in control group	*I*^2^ (%)	P Value for heterogeneity	OR 95% CI	Overall effect	Z /P Value	Fragility index	Fragility quotient
Reduced adverse events	8/26	504/	3967	716/3925	89	0.000	0.54 [0.33, 0.89]	2.42/ < 0.015	6.5 (2–21.25)*	0.024 (0.011–0.073)
Clinical improvement rate	15/21	1354/1966	1059/1929	44.3	0.016	1.36 [1.15, 1.60]	3.63/0.000	2 (0.5–9)*	0.022 (0.001–0.045)	
Viral nucleic acid negative rate	10/14	616/889	243/843	69.7	0.000	1.97 [1.38, 2.80]	3.76/0.000	4 (1.75–13.5)	0.052 (0.019–0.155)	
Clinical deterioration rate	11/16	204/1439	385/	1405	56.2	0.003	0.4 [0.28, 0.58]	4.88/0.000	2 (1–3.75)*	0.014 (0.007–0.020)
Mortality	7/8	56/277	85/255	67.1	0.003	0.55 [0.26, 1.18]	1.54/0.124	1 (0.5–4.5)*	0.032 (0.002–0.071)	
Needing hospitalization or ICU	4/4	45/326	105/	313	67	0.028	0.37 [0.17, 0.80]	2.52/0.012	1 (0–1.5)*	0.041 (0.001–0.063)
Rate of escalation respiratory support	5/9	59/769	156/	770	71.2	0.001	0.29 [0.13, 0.67]	2.90/0.004	2 (1–8)	0.018 (0.007–0.041)
Severe malfunction of key organs	9/12	104/812	274/	799	74.2	0.000	0.29 [0.16, 0.53]	3.96/0.000	1 (0.25–3.75)*	0.025 (0.020–0.051)
Rate of symptoms control	5/10	41/427	132/	427	51.5	0.029	0.19 [0.15, 0.55]	3.79/0.000	2.5 (1–4.5)	0.029 (0.020–0.044)

OR, odds ratio;ICU,intensive care unit. ^*^FIs of outcomes about adverse events were higher than those about improvement rate, clinical deterioration rate, prognosis and aggravated function of key organs (T = 23.887, 30.02, 31.723, 35.090, P = 0.02,0.007,0.015,0.004, respectively)

### The Overall Distribution of the FIs and FQs of the Significant Dichotomous Outcomes

**Fig. 2:**
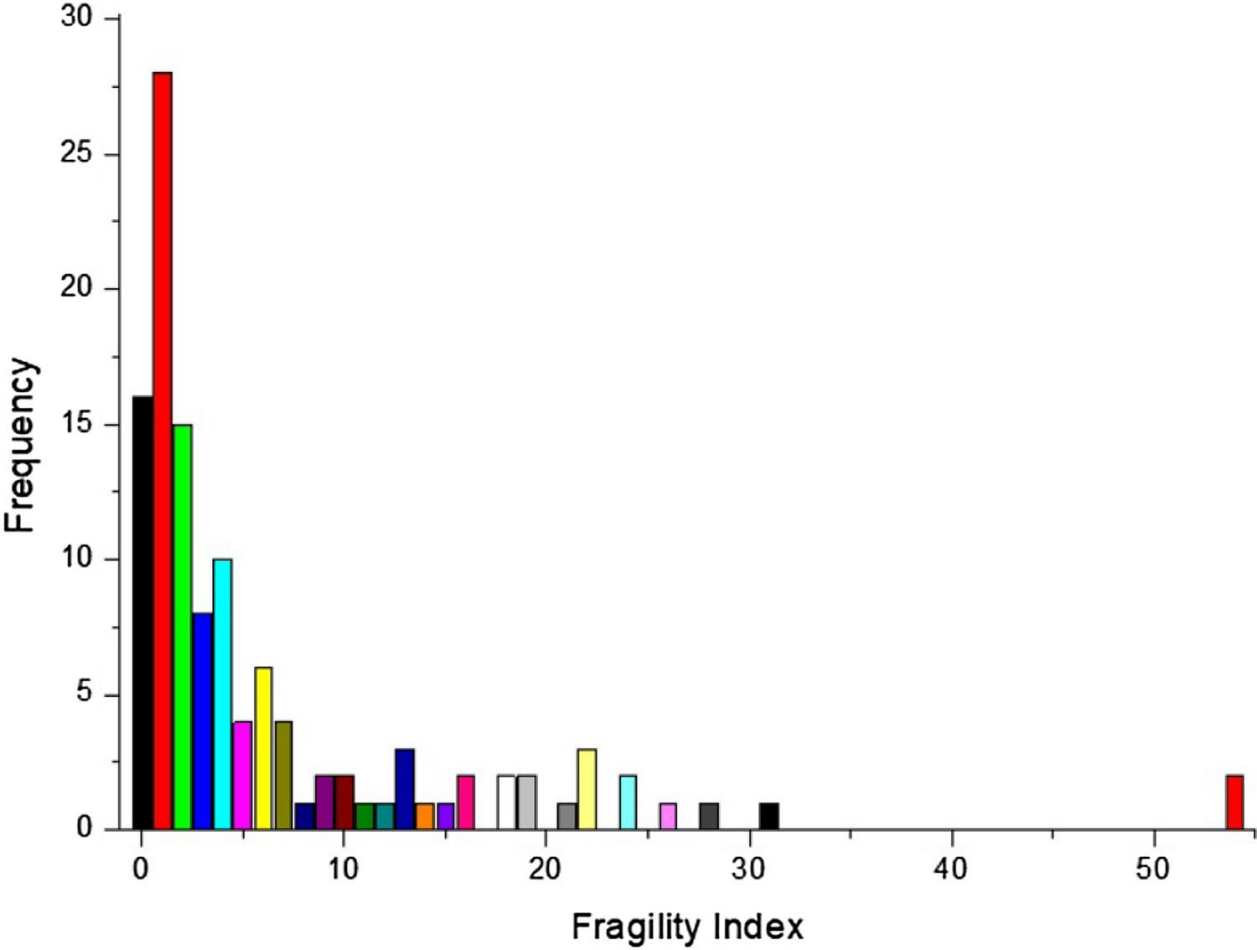
Frequency distribution of fragility index of the significant dichotomous outcomes. The minimum value of fragility index was 0 and the maximum value was 54,a FI of 0 indicated P value became > 0.05 by using Fisher exact test instead of chi-square test without altering the numbers of events

The distribution of the FIs was abnormal with median 3 and IQR 1–7 (P = 0.000, Fig. 2); the minimum value of the FI was 0 and the maximum value was 54 in one outcome. Twenty-eight outcomes (23.33%, 28/120) had an FI of 1, whereas 61 (50.83%, 61/120) had an FI of greater than or equal to 3; additionally, there were 16 (13.33%, 16/120) outcomes with FIs equal to 0, which signified that the previous significant difference became nonsignificant when analyzed by Fisher’s test. Twenty-five (50%) RCTs reported more than one outcome with a significant difference and hence, we obtained more than one FI in these trials. The FQs were distributed abnormally with a median of 0.0223 and an IQR of 0.009–0.054 (P = 0.0001).

### The Impact Factors of FI Presented By Categorical Data

The FIs in the RCTs registered in ClinicalTrail.gov (median, 4; IQR, 1–10.75) were significantly higher than those in the RCTs registered in other registration domains (median, 2; IQR, 1–4; T = – 3.079, P = 0.002).There were significant differences of the FIs among the three kinds of patients with different severity (T = 16.667, P = 0.0001). The FIs in outpatients were higher than those in the hospitalized severe patients [(median, 5; IQR, 2–18) versus (median, 1; IQR, 0–3)] and patients with various severity [(median, 5; IQR, 2–18) versus (median, 2; IQR, 1–7.5)], and T = 32.218, – 17.945, P = 0.000, 0.027, respectively. The robustness of the outcome was affected by the intervention method, FIs among outcomes with different interventions were statistically significantly different (T = 23.107, P = 0.001); furthermore, pairwise comparisons revealed that the FIs of nonspecific immunostimulants (median, 1; IQR, 0–1) were dramatically lower than the antiviral treatments (median, 4; IQR, 2–9.75; T = – 46.144; P = 0.000), local traditional medicine (median, 3; IQR, 1–13; T = -44.815; P = 0.002), anticoagulation (median, 6; IQR, 0–12; T = – 35.341; P = 0.029) and specific immunosuppressants (median, 4.5; IQR, 1–18.25; T = – 45.624; P = 0.000). The control strategies did not affect the value of the FI (T = 2.767, P = 0.251).

Outcome status, primary or secondary, whether the outcome suggested patients benefiting from the intervention or not did not impact the FI (T = 0.701 and P = 0.483, T = 681 and P = 0.496, respectively). The other characteristics, such as whether the RCTs were being performed in multicentric setting, with a JADAD score of, and employed RCT concealment did not influence the FI, i.e., the robustness (T = – 0.481, – 0.539,4.489 and P = 0.63, 0.59, 0.106, respectively). There was no significant difference between/among the subgroups according to the other categorical characteristics (P > 0.05), as shown in Table 2.

**Table 2:** Correlation of categorical trial characteristics with FI and FQ

Characteristics	Fragility Index (n = 120)			Fragility Quotient (n = 120)			N/n^@^
	Median (IQR)	T	P	Median (IQR)	T	P	
Registration		– 3.079	0.002		– 0.524	0.60	
ClinicalTrail.gov	4 (1–10.75)			0.022 (0.01–0.057)			21/64
The others	2 (1–4)			0.024 (0.004–0.051)			29/56
Multi-center		– 0.481	0.630		– 1.433	0.152	
Yes	2 (1–6)			0.026 (0.011–0.058)			21/45
No	3 (1–8)			0.015 (0.007–0.049)			29/75
JADAD score		– 0.539	0.590		– 0.761	0.441	
≤ 2	3 (1.5–13.5)			0.032 (0.005–0.068)			8/17
≥ 3	3 (1–7)			0.021 (0.01–0.055)			42/103
Randomization concealment	(– )	4.489	0.106		18.525	0.000	
0	4 (2–22)			0.042 (0.032–0.199)			5/11
1	3 (1–11)			0.026 (0.011–0.067)			23/65
2	2 (1–6)			0.012 (0.004–0.026)			22/44
Patients^×^							
Outpatients	5 (2–18)	16.667	0.000	0.029 (0.014–0.067)	4.265	0.119	11/47
Hospitalized severe patients	1 (0–3)			0.027 (0.000–0.051)			19/32
Hospitalized patients	2 (1–7.5)			0.018 (0.008–0.034)			20/41
Intervention		23.107	0.001		9.394	0.153	
Nonspecific immunostimulants^#^	1 (0–1)			0.010 (0.000–0.043)			8/13
Nonspecific immunosuppressants^*^	2 (1–4)			0.020 (0.010–0.048)			11/19
Antiviral treatment^#*^	4 (2–9.75)			0.029 (0.016–0.071)			10/24
Breathing support	1.5 (1–3.75)			0.016 (0.090–0.048)			3/8
Traditional medicine^#^	3 (1–13)			0.036 (0.011–0.058)			6/11
Anticoagulation^#^	6 (0–12)			0.010 (0.000–0.020)			3/7
Specific immunosuppressants^#*^	4.5 (1–18.25)			0.026 (0.009–0.068)			9/38
Control strategy		2.767	0.251		2.887	0.23	
Only standard treatment	3 (1–5)			0.030 (0.010–0.062)			25/47
Placebo	4 (1–14.5)			0.022 (0.009–0.058)			16/53
Non-placebo control methods	2 (1–6)			0.016 (0.009–0.032)			9/20
Outcome status		0.701	0.483		– 0.061	0.951	
Primary	2 (1–5.5)			0.021 (0.010–0.057)			30/43
Secondary	3 (1–9)			0.025 (0.010–0.055)			20/79
Outcomes benefiting from intervention		0.681	0.496		– 0.102	0.919	
Yes	2 (1–7.25)			0.024 (0.009–0.055)			44/106
No	5 (1–8.25)			0.020 (0.010–0.051)			6/14

FI, fragility index; FQ, fragility quotient. ^@^N, number of studies, n, number of outcomes; ^×^ The FIs in outpatients were higher than those in the hospitalized severe patients and patients with various severity [T = 32.218, – 17.945, P = 0.000,0.027,respectively]; ^#^Further pairwise comparisons showed the FIs of nonspecific immunostimulants were dramatically lower than antiviral treatments (T = – 46.144, P = 0.000), local traditional medicine (T = – 44.815, P = 0.002), anticoagulation (T =  – 35.341, P = 0.029) and specific immunosuppressants (T = – 45.624, P = 0.000);^*^ indicated FIs of nonspecific immunosuppressants were lower than those of antiviral treatments (T = – 22.033, P = 0.037)and specific immunosuppressants (T = – 21.53, P = 0.026); ^&^FIs of viral nucleic acid negative rate were higher than those of aggravated function of key organs (T = 27.345, P = 0.047)

### The Impact Factors of FI Presented By Continuous Data

**Fig. 3:**
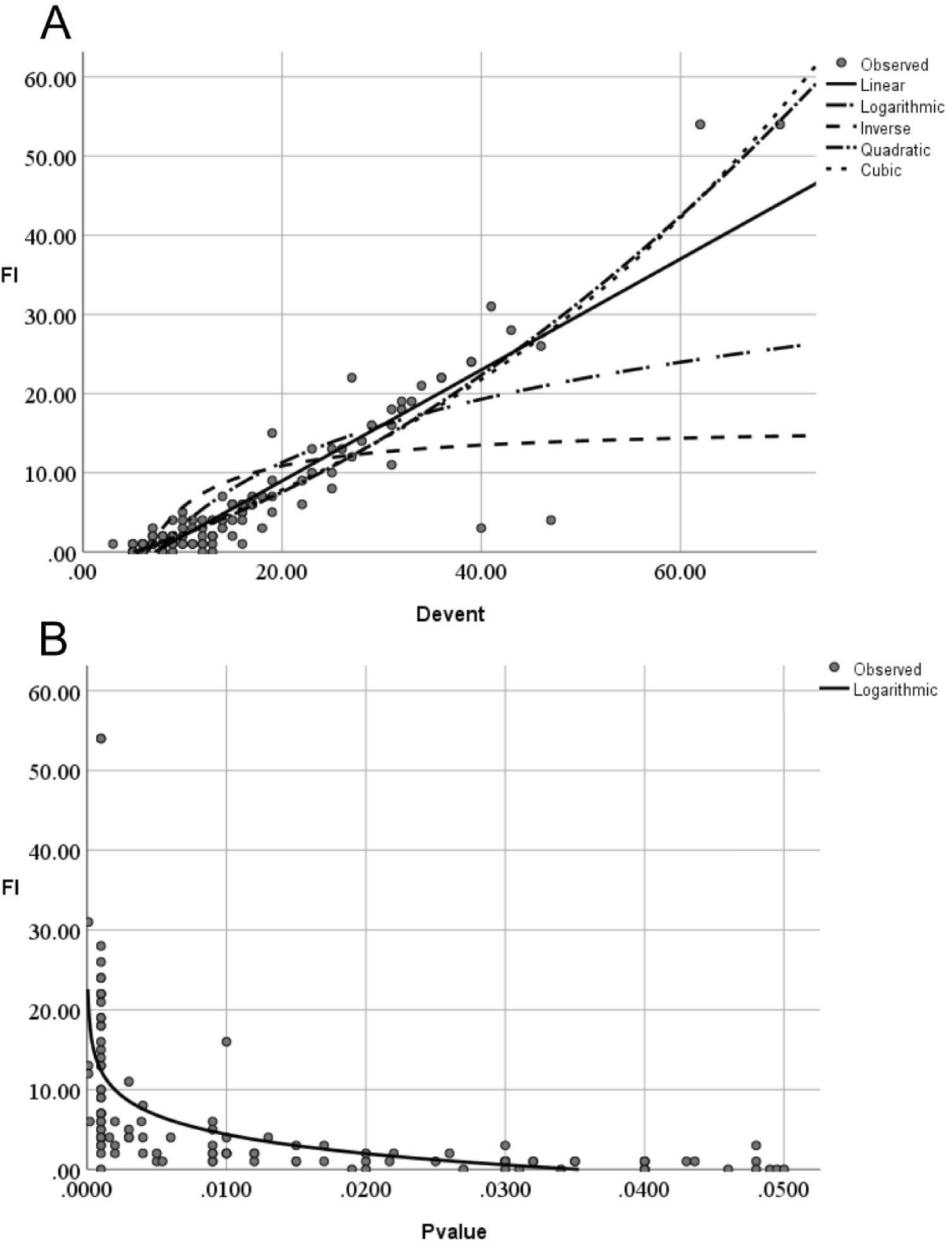
Correlation of quantitative characteristics of RCT with fragility index. FI, fragility index; Devent, absolute difference of events between intervention and control groups. Panel A Correlation of Devent with FI. The maximal R square was generated when the relationship between FI and Devent was fitted with cubic model, R square = 0.848, P = 0.000; Panel B Correlation of P value with FI. A strong negative logarithmic relationship between the FI and the P value with R square = 0.366, P = 0.000

There was a moderate positive correlation between FI and the characteristics presented by continuous data, such as sample size, in the intervention/control group, total sample size, events in the control group, and total events with the Spearman correlation coefficient equal to 0.513, 0.503, 0.528, 0.466, and 0.446, respectively; however, the maximal R squares were low, between 0.180 and 0.257, when curve estimations were performed. We observed a good cubic relationship between the FI and the absolute difference of the events between the two groups with adjusted R-square 0.848 (T = 215.828, P = 0.0001, Fig. 3A); the Spearman correlation coefficient was 0.865 with P = 0.0001, which indicated a strong positive correlation between the FI and the absolute difference in the events. We also found a strong negative logarithmic relationship between the FI and the P value with Spearman correlation coefficient – 0.834 (Fig. 3B). The correlation between the FI and the other characteristics was weak; detailed information is presented in Table 3.

**Table 3:** Correlation of quantitative trial characteristics with fragility index and fragility quotient

Characteristics	Fragility Index (n = 120)	Fragility Quotient (n = 120)				
	Spearman correlation coefficient	P value	R square	Spearman correlation coefficient	P value	R square
Events in intervention group	0.176	0.055	0.031*	0.029	0755	0.022*
Sample size in intervention group	0.513	0.000	0.194*	– 0.015	0.868	0.025*
Events in control group	0.466	0.000	0.257*	0.241	0.008	0.097*
Sample size in control group	0.503	0.000	0.180*	– 0.029	.0.756	0.019*
Total events	0.446	0.000	0.254*	0.209	0.022	0.131*
Total sample size	0.528	0.000	0.198*	– 0.013	0.889	0.023*
Absolute difference of events between the two groups	0.865	0.000	0.848*	0.573	0.000	0.456*
Dropouts in intervention group	– 0.028	0.759	0.026*	– 0.175	0.057	0.024*
Dropouts in control group	0.351	0.000	0.118*	0.106	0.248	0.008*
Total dropouts	0.293	0.001	0.073*	0.039	0.674	0.007*
P value	– 0.834	0.000	0.366#	– 0.650	0.000	0.268#

^*^ Fitting to cubic curve, #Fitting to logarithmic curve

## Discussion

To our knowledge, this was the first study adopting FI and FQ as simplified and intuitive metrics to quantify the robustness of significant dichotomous results in RCTs on COVID-19. In this study, we found that the FIs were small, which indicated that the robustness was still fragile in the reported outcome with a significant difference. FIs varied in the different outcomes of interest and some characteristics of the RCTs, such as registration, various patient populations, and intervention strategies, which affected the value of the FI and the robustness of the result.

In this particular difficult era of the world disturbed by the SARS-CoV-2, the people were overwhelmed with unfavorable emotions, such as anxiety, depression, and insomnia [25], and hence, were anxious for an effective treatment for COVID-19. The current commonly used criterion for a significant result is a P-value lower than the set cut-off point (for example P < 0.05). However, it is not perfect [26], especially, when the actual P-value is close to the cut-off, usually 0.05. In this condition, decreasing a few or even one event in the group with larger number of events or increasing a few events in the group with smaller number of events would transform the “significant” result to an insignificant one, which makes the result very weak for reliability. As shown in this study, up to 49.17% of the outcomes were found with FI no more than 2, which demonstrated that the significant findings could be overturned by shifting two participants from the nonevents to the events; thus, the evidence of the significant findings was very fragile, especially in the RCTs in which strict blindness strategies were not adopted in performance and data collection.

In this study, we observed that the median of outcomes was 3, which was similar to the findings in the other subspecialties, such as critical care medicine [19], trauma [17], anesthesiology [20], sports medicine [27], and spine surgery [28]; however, the median was lower than 13 (IQR, 5–26) in the cardiovascular RCTs [29]. It is noteworthy that interpreting the FI combined with the sample size would be better [30]; thus, we used the FQ to evaluate the robustness further and found that the median of the FQ (median 0.0223 and IQR 0.009–0.054) in this study was higher than 0.0042 (IQR 0.0020–0.0110) in the cardiovascular medicine subspecialty [29]. The higher FQ in this study indicated that the significant outcome would be nonsignificant if 2.2 patients per 100 experienced a reverse event, which was 5.5 times of merely 0.4 patients per 100 in the aforementioned cardiovascular medicine [29]. The larger FQ demonstrated more stability of the findings, although the FIs were relatively low. Generally, we considered that the evidence of the dichotomous outcomes with significant difference was fragile; thus, it is necessary to increase the sample size in further RCTs to increase the robustness. In clinical practice, we recommend to calculate the FI and adopt it as a quantitative metric to evaluate the strength of the evidence when we would select a new therapeutic option.

The correlation analysis indicated that FI might be affected by some characteristics of the RCTs such as various patient populations, the outcomes of interest, and different interventions. The outcomes in the outpatients possessed the strongest robustness while the result in the hospitalized severe patients acquired the most fragile one. We considered these were associated with the fact that most of the outcomes to be used for evaluating the effect of the treatments in the outpatients were about the symptom control and reduction in the adverse events, while outcomes of interest in the hospitalized severe patients were largely relevant to mortality and the extubation rate. It might be easy to obtain an event regarding symptom control or prevent an adverse event, while difficult to reduce a death, which was consistent with the nonsignificant pooled result of mortality and significant pooled results of the other outcomes (Table 1). Additionally, as shown in Table 2, 11 RCTs in the outpatients reported 47 significant outcomes (15 adverse events, 10 symptoms) while 19 RCTs in the hospitalized severe patients only reported 32 outcomes (mortality, extubation rate) with dramatic difference. This might indirectly confirm that there were few options for improving the prognosis of patients with severe COVID-19 based on current research. In addition, the robustness also varied in the RCTs with different intervention strategies. The median of the FI in antiviral treatments (including drugs, such as remdesivir, sofosbuvir, and favipiravir, and convalescent plasma therapy) was in the upstream of all the interventions, which indicated that the studies of antiviral therapy reported significant outcomes, which were believable although most antiviral drugs had demonstrated mixed results or even no beneficial effects [31]. Besides the antiviral treatments, immunomodulatory therapy demonstrated great expectations [32]. The results in this study indicated that FIs varied in different immunomodulatory therapies. Median of the FIs in the nonspecific immunostimulants, such as interferon and Mycobacterium vaccae, was the smallest and the evidence was the most fragile compared with immunosuppressants including nonspecific (hydroxychloroquine, glucocorticoid, colchicine) or specific ones (tofacitinib, bocilizumab). This could be explained by the immune features of the cytokine storm; in the early stage of the infection the secretion of interferon was delayed, whereas in the late stage, pro-inflammatory cytokines were excessively secreted [33]. Immunostimulants could be urgently needed in the early stage and immunosuppressants could work better in the late stage [34]. However, the RCTs that adopted immunostimulants were performed in the hospitalized patients, even some of them were severe cases at late stage [35–39]. The higher FI of specific immunosuppressants supported the judgment that new generation cytokine-targeted therapies, such as tofacitinib and tocilizumab, could be the most promising drugs [34].

FI was also impacted by some characteristics described by continuous data. There was a positive cubic correlation between FI and the absolute difference of events between the intervention and control group, which was understandable. The larger the absolute difference between the groups, the more the number of events needed to reverse the significant outcomes. The total sample size was also an important impact factor with a high correlation coefficient, which was consistent with the previous studies in critical care medicine [18, 19] and spine surgery [28], but inconsistent with a study in heart failure [40] and recently published reports in patients with solid cancers [41]. The relationship between FI and P-value was negative logarithmic correlation; the smaller the P value the larger the FI. In fact, both the FI and P-value were metrics to evaluate the difference between the group [42], FI was more straightforward and convenient for clinicians to understand but only used for significant binary outcomes, whereas the P-value was applied more extensively but it was more obscure.

In addition, the RCTs registered in ClinicalTrial.gov were prone to report outcomes with higher FI compared with those in the other registrations, which might be associated with the fact that most of the trials registered in agency registry were performed in developing countries, the design and performance of trials could be slightly different in developed nations [43, 44]. This study tried to assist clinicians in the interpretation of the significant outcomes more precisely with a quantitative metric; however, we did not aim to question the findings researched by the numerous great unsung heroes.

There were some limitations to this study. First, we did not consider the continuous outcomes although they promoted recognition of SARS-COV-2 because FI itself could only be used for binary outcomes. Second, we had to blend some studies with similar characteristics together for a feasible analysis owing to paucity of the RCTs with the completely same features. Third, there was no way to include all of the RCTs on the treatment of COVID-19, because COVID-19 had not been elucidated fully and articles from RCTs have been publishing continuously. However, the embarrassing scene did not affect the purpose of the present study, to remind the reader to evaluate the robustness of a significant binary outcome on the treatment of COVID-19 with a quantitative metric, FI. Fourth, in the present study, a quantitative assessment tool, JADAD scale, was adopted to estimate the methodological quality of RCTs, and to analyze the relationship between total score of JADAD scale (continuous data) and the FI. However, JADAD scale might underestimate the quality of open RCTs in which blinding to the participants or staff was impossible, for instance, receiving breathing support strategies or not. Finally, the results could only be used as a clear metric of the robustness for a binary outcome with significant difference; however, it could not arrive at a conclusion on the effect of an intervention strategy.

## Conclusion

The robustness of significant dichotomous outcomes was still fragile in the RCTs on the treatment of patients with COVID-19. FIs were mainly affected by the outcomes of interest, patients, interventions, P value and absolute difference of events between the groups. The robustness of the outcomes in the outpatients, specific immunosuppressant, and antiviral treatment was stronger. Thus, we recommend the routine report of the FI and FQ as quantitative metrics to assist the readers in better interpretation of a binary outcome with significant difference.

## Supplementary Information

Below is the link to the electronic supplementary material.

Supplementary file1 (PDF 225 kb)

## Data Availability

The datasets used and/or analyzed during the current study available from the corresponding author (Q.L.) on reasonable request (https://www.researchgate.net/profile/Qi-Liu-169/publications).
