# Supplementary material for: Robustness of Significant Dichotomous Outcomes in Randomized Controlled Trials in the Treatment of Patients with COVID-19: A Systematic Analysis
Source: Intensive Care Res. 2023 Jan 12;3(1):38–49. doi: 10.1007/s44231-022-00027-y (PMC9836340; doi:10.1007/s44231-022-00027-y)
Supplement: Supplementary file 1 — Supplementary file1 (PDF 225 kb) [file 44231_2022_27_MOESM1_ESM.pdf]

**Supplementary materials**

## Robustness of Significant Dichotomous Outcomes in Randomized Controlled Trials in the Treatment of Patients with COVID-19: A Systematic Analysis

**Supplementary Table S1. References of the included studies**

| | |
|---|---|
| Iran[1-10] | Brazil[11-18], United Kingdom[19,20], United States[21-23], China[24-31], India [32-36], Canada [37], Italy [38,39], and the other countries[43-48], additionally, two RCTs were completed in multiple countries[49,50] |


| | |
|---|---|
| 1 | Davoudi-Monfared, E. *et al.* A Randomized Clinical Trial of the Efficacy and Safety of Interferon beta-1a in Treatment of Severe COVID-19. *Antimicrob Agents Chemother* 64, doi:10.1128/AAC.01061-20 (2020). |
| 2 | Edalatifard, M. *et al.* Intravenous methylprednisolone pulse as a treatment for hospitalised severe COVID-19 patients: results from a randomised controlled clinical trial. *Eur Respir J* 56, doi:10.1183/13993003.02808-2020 (2020). |
| 3 | Eslami, G. *et al.* The impact of sofosbuvir/daclatasvir or ribavirin in patients with severe COVID-19. *J Antimicrob Chemother* 75, 3366-3372, doi:10.1093/jac/dkaa331 (2020). |
| 4 | Gharebaghi, N., Nejadrahim, R., Mousavi, S. J., Sadat-Ebrahimi, S. R. & Hajizadeh, R. The use of intravenous immunoglobulin gamma for the treatment of severe coronavirus disease 2019: a randomized placebo-controlled double-blind clinical trial. *BMC Infect Dis* 20, 786, doi:10.1186/s12879-020-05507-4 (2020). |
| 5 | Rahmani, H. *et al.* Interferon beta-1b in treatment of severe COVID-19: A randomized clinical trial. *Int Immunopharmacol* 88, 106903, doi:10.1016/j.intimp.2020.106903 (2020). |
| 6 | Ranjbar, K. *et al.* Methylprednisolone or dexamethasone, which one is superior corticosteroid in the treatment of hospitalized COVID-19 patients: a triple-blinded randomized controlled trial. *BMC Infect Dis* 21, 337, doi:10.1186/s12879-021-06045-3 (2021). |
| 7 | Roostaei Firozabad, A. *et al.* Efficacy and safety of Levamisole treatment in clinical presentations of non-hospitalized patients with COVID-19: a double-blind, randomized, controlled trial. *BMC Infect Dis* 21, 297, doi:10.1186/s12879-021-05983-2 (2021). |
| 8 | Roozbeh, F. *et al.* Sofosbuvir and daclatasvir for the treatment of COVID-19 outpatients: a double-blind, randomized controlled trial. *J Antimicrob* |

*Chemother* 76, 753-757, doi:10.1093/jac/dkaa501 (2021).

9 Sadeghi, A. *et al.* Sofosbuvir and daclatasvir compared with standard of care in the treatment of patients admitted to hospital with moderate or severe coronavirus infection (COVID-19): a randomized controlled trial. *J Antimicrob Chemother* 75, 3379-3385, doi:10.1093/jac/dkaa334 (2020).

10 Solaymani-Dodaran, M. *et al.* Safety and efficacy of Favipiravir in moderate to severe SARS-CoV-2 pneumonia. *Int Immunopharmacol* 95, 107522, doi:10.1016/j.intimp.2021.107522 (2021).

11 Guimaraes, P. O. *et al.* Tofacitinib in Patients Hospitalized with Covid-19 Pneumonia. *N Engl J Med* 385, 406-415, doi:10.1056/NEJMoa2101643 (2021).

12 Lemos, A. C. B. *et al.* Therapeutic versus prophylactic anticoagulation for severe COVID-19: A randomized phase II clinical trial (HESACOVID). *Thromb Res* 196, 359-366, doi:10.1016/j.thromres.2020.09.026 (2020).

13 Lopes, M. I. *et al.* Beneficial effects of colchicine for moderate to severe COVID-19: a randomised, double-blinded, placebo-controlled clinical trial. *RMD Open* 7, doi:10.1136/rmdopen-2020-001455 (2021).

14 Lopes, R. D. *et al.* Therapeutic versus prophylactic anticoagulation for patients admitted to hospital with COVID-19 and elevated D-dimer concentration (ACTION): an open-label, multicentre, randomised, controlled trial. *Lancet* 397, 2253-2263, doi:10.1016/S0140-6736(21)01203-4 (2021).

15 McCoy, J. *et al.* Proxalutamide Reduces the Rate of Hospitalization for COVID-19 Male Outpatients: A Randomized Double-Blinded Placebo-Controlled Trial. *Front Med (Lausanne)* 8, 668698, doi:10.3389/fmed.2021.668698 (2021).

16 Rea-Neto, A. *et al.* An open-label randomized controlled trial evaluating the efficacy of chloroquine/hydroxychloroquine in severe COVID-19 patients. *Sci Rep* 11, 9023, doi:10.1038/s41598-021-88509-9 (2021).

17 Silveira, M. A. D. *et al.* Efficacy of Brazilian green propolis (EPP-AF(R)) as an adjunct treatment for hospitalized COVID-19 patients: A randomized, controlled clinical trial. *Biomed Pharmacother* 138, 111526, doi:10.1016/j.biopha.2021.111526 (2021).

18 Veiga, V. C. *et al.* Effect of tocilizumab on clinical outcomes at 15 days in patients with severe or critical coronavirus disease 2019: randomised controlled trial. *BMJ* 372, n84, doi:10.1136/bmj.n84 (2021).

19 Monk, P. D. *et al.* Safety and efficacy of inhaled nebulised interferon beta-1a (SNG001) for treatment of SARS-CoV-2 infection: a randomised, double-blind, placebo-controlled, phase 2 trial. *Lancet Respir Med* 9, 196-206, doi:10.1016/S2213-2600(20)30511-7 (2021).

20 Ramakrishnan, S. *et al.* Inhaled budesonide in the treatment of early COVID-19 (STOIC): a phase 2, open-label, randomised controlled trial. *Lancet*

*Respir Med* 9, 763-772, doi:10.1016/S2213-2600(21)00160-0 (2021).
21 Lenze, E. J. *et al.* Fluvoxamine vs Placebo and Clinical Deterioration in Outpatients With Symptomatic COVID-19: A Randomized Clinical Trial. *JAMA* 324, 2292-2300, doi:10.1001/jama.2020.22760 (2020).
22 Lanzoni, G. *et al.* Umbilical cord mesenchymal stem cells for COVID-19 acute respiratory distress syndrome: A double-blind, phase 1/2a, randomized controlled trial. *Stem Cells Transl Med* 10, 660-673, doi:10.1002/sctm.20-0472 (2021).
23 Huang, Y. H. & Huang, J. T. Use of chlorhexidine to eradicate oropharyngeal SARS-CoV-2 in COVID-19 patients. *J Med Virol* 93, 4370-4373, doi:10.1002/jmv.26954 (2021).
24 Li, L. *et al.* Effect of Convalescent Plasma Therapy on Time to Clinical Improvement in Patients With Severe and Life-threatening COVID-19: A Randomized Clinical Trial. *JAMA* 324, 460-470, doi:10.1001/jama.2020.10044 (2020).
25 Cao, Y. *et al.* Ruxolitinib in treatment of severe coronavirus disease 2019 (COVID-19): A multicenter, single-blind, randomized controlled trial. *J Allergy Clin Immunol* 146, 137-146 e133, doi:10.1016/j.jaci.2020.05.019 (2020).
26 Wu, C. N. *et al.* High-flow nasal-oxygenation-assisted fibreoptic tracheal intubation in critically ill patients with COVID-19 pneumonia: a prospective randomised controlled trial. *Br J Anaesth* 125, e166-e168, doi:10.1016/j.bja.2020.02.020 (2020).
27 Wang, J. B. *et al.* Exploring an Integrative Therapy for Treating COVID-19: A Randomized Controlled Trial. *Chin J Integr Med* 26, 648-655, doi:10.1007/s11655-020-3426-7 (2020).
28 Xu, X. *et al.* Efficacy and safety of Reduning injection in the treatment of COVID-19: a randomized, multicenter clinical study. *Ann Palliat Med* 10, 5146-5155, doi:10.21037/apm-20-2121 (2021).
29 Lin, Y. R. *et al.* Mycobacterium vaccae Nebulization in the Treatment of COVID-19: A Randomized, Double-Blind, Placebo-Controlled Trial. *J Aerosol Med Pulm Drug Deliv* 34, 108-114, doi:10.1089/jamp.2020.1628 (2021).
30 Hu, K. *et al.* Efficacy and safety of Lianhuaqingwen capsules, a repurposed Chinese herb, in patients with coronavirus disease 2019: A multicenter, prospective, randomized controlled trial. *Phytomedicine* 85, 153242, doi:10.1016/j.phymed.2020.153242 (2021).
31 Wang, D. *et al.* Tocilizumab in patients with moderate or severe COVID-19: a randomized, controlled, open-label, multicenter trial. *Front Med* 15, 486-494, doi:10.1007/s11684-020-0824-3 (2021).
32 Pandit, A. *et al.* Efficacy and safety of pegylated interferon alfa-2b in moderate COVID-19: A phase II, randomized, controlled, open-label study. *Int J Infect Dis* 105, 516-521, doi:10.1016/j.ijid.2021.03.015 (2021).

33 Devpura, G. *et al.* Randomized placebo-controlled pilot clinical trial on the efficacy of ayurvedic treatment regime on COVID-19 positive patients. *Phytomedicine* 84, 153494, doi:10.1016/j.phymed.2021.153494 (2021).

34 Thakar, A. *et al.* Chloroquine nasal drops in asymptomatic & mild COVID-19: An exploratory randomized clinical trial. *Indian J Med Res* 153, 151-158, doi:10.4103/ijmr.IJMR_3665_20 (2021).

35 Soin, A. S. *et al.* Tocilizumab plus standard care versus standard care in patients in India with moderate to severe COVID-19-associated cytokine release syndrome (COVINTOC): an open-label, multicentre, randomised, controlled, phase 3 trial. *Lancet Respir Med* 9, 511-521, doi:10.1016/S2213-2600(21)00081-3 (2021).

36 Shah, M. *et al.* Safety and efficacy of ozone therapy in mild to moderate COVID-19 patients: A phase 1/11 randomized control trial (SEOT study). *Int Immunopharmacol* 91, 107301, doi:10.1016/j.intimp.2020.107301 (2021).

37 Feld, J. J. *et al.* Peginterferon lambda for the treatment of outpatients with COVID-19: a phase 2, placebo-controlled randomised trial. *Lancet Respir Med* 9, 498-510, doi:10.1016/S2213-2600(20)30566-X (2021).

38 Rocco, P. R. M. *et al.* Early use of nitazoxanide in mild COVID-19 disease: randomised, placebo-controlled trial. *Eur Respir J* 58, doi:10.1183/13993003.03725-2020 (2021).

39 Grieco, D. L. *et al.* Effect of Helmet Noninvasive Ventilation vs High-Flow Nasal Oxygen on Days Free of Respiratory Support in Patients With COVID-19 and Moderate to Severe Hypoxemic Respiratory Failure: The HENIVOT Randomized Clinical Trial. *JAMA* 325, 1731-1743, doi:10.1001/jama.2021.4682 (2021).

40 Koshak, A. E. *et al.* Nigella sativa for the treatment of COVID-19: An open-label randomized controlled clinical trial. *Complement Ther Med* 61, 102769, doi:10.1016/j.ctim.2021.102769 (2021).

41 Rosen, J. *et al.* Awake prone positioning in patients with hypoxemic respiratory failure due to COVID-19: the PROFLO multicenter randomized clinical trial. *Crit Care* 25, 209, doi:10.1186/s13054-021-03602-9 (2021).

42 Gonzalez-Ochoa, A. J. *et al.* Sulodexide in the Treatment of Patients with Early Stages of COVID-19: A Randomized Controlled Trial. *Thromb Haemost* 121, 944-954, doi:10.1055/a-1414-5216 (2021).

43 Supady, A. *et al.* Cytokine adsorption in patients with severe COVID-19 pneumonia requiring extracorporeal membrane oxygenation (CYCOV): a single centre, open-label, randomised, controlled trial. *Lancet Respir Med* 9, 755-762, doi:10.1016/S2213-2600(21)00177-6 (2021).

44 Aref, Z. F. *et al.* Clinical, Biochemical and Molecular Evaluations of Ivermectin Mucoadhesive Nanosuspension Nasal Spray in Reducing Upper

Respiratory Symptoms of Mild COVID-19. *Int J Nanomedicine* 16, 4063-4072, doi:10.2147/IJN.S313093 (2021).

45 Samaha, A. A. *et al.* Effects of a Single Dose of Ivermectin on Viral and Clinical Outcomes in Asymptomatic SARS-CoV-2 Infected Subjects: A Pilot Clinical Trial in Lebanon. *Viruses* 13, doi:10.3390/v13060989 (2021).

46 Mahmud, R. *et al.* Ivermectin in combination with doxycycline for treating COVID-19 symptoms: a randomized trial. *J Int Med Res* 49, 3000605211013550, doi:10.1177/03000605211013550 (2021).

47 Deftereos, S. G. *et al.* Effect of Colchicine vs Standard Care on Cardiac and Inflammatory Biomarkers and Clinical Outcomes in Patients Hospitalized With Coronavirus Disease 2019: The GRECCO-19 Randomized Clinical Trial. *JAMA Netw Open* 3, e2013136, doi:10.1001/jamanetworkopen.2020.13136 (2020).

48 Libster, R. *et al.* Early High-Titer Plasma Therapy to Prevent Severe Covid-19 in Older Adults. *N Engl J Med* 384, 610-618, doi:10.1056/NEJMoa2033700 (2021).

49 Skipper, C. P. *et al.* Hydroxychloroquine in Nonhospitalized Adults With Early COVID-19 : A Randomized Trial. *Ann Intern Med* 173, 623-631, doi:10.7326/M20-4207 (2020).

50 Beigel, J. H. *et al.* Remdesivir for the Treatment of Covid-19 - Final Report. *N Engl J Med* 383, 1813-1826, doi:10.1056/NEJMoa2007764 (2020).

**Supplementary Table S2.Characteristics of the included studies ,fragility index and JADAD scoring in this studies (n = 50)**

| Study | Participants. | Interventions | Control | Sample | | Dropout | | Outcomes [ (fragility index),(event in intervention/event in control)] | JADAD score |
|---|---|---|---|---|---|---|---|---|---|
| | | | | I | C | I | C | | |
| Skipper 2020 | Nonhospitalized adults with early COVID-19 | Hydroxychloroquine | Placebo | 212 | 211 | 13 | 13 | Incidence of medication adverse reactions[(26),(92/46)] | 5 |
| Li 2020 | Hospitalised severe COVID-19 patients | Convalescent plasma therapy | Standard treatment alone | 52 | 51 | 0 | 0 | Viral nucleic acid negative rate at 24h [(4),(21/6)],48h [(15),(32/13)],72h [(13),(41/15)];<br>Viral nucleic acid negative rate at 72h for severe disease subgroup[(3),(19/7)];<br>Clinical improvement rate at day 14 for severe subgroup [(1),(14/6)] | 3 |
| Lenze 2020 | Outpatients with symptomatic COVID-19 | Fluvoxamine | Placebo | 80 | 72 | 0 | 0 | Clinical deterioration rate [(1),(0/6)];<br>None clinical deterioration rate [(3),(80/66)];<br>The rate of any nonzero value of clinical status on 7-point scale [(1),(0/6)] | 5 |
| Cao 2020 | Hospitalised severe COVID-19 patients | Ruxolitinib | Placebo | 20 | 21 | 0 | 0 | Rate of improvement on the follow-up chest CT scans at day [(0),(18/13)] | 5 |
| Beigel 2020 | Hospitalized patients with COVID-19 | Remdesivir | Placebo | 541 | 521 | 0 | 0 | Recovery rate [(4),(399/352)] | 3 |

| | | | | | | | | | |
|---|---|---|---|---|---|---|---|---|---|
| Lemos 2020 | Hospitalised severe COVID-19 patients | Therapeutic enoxaparin | Prophylactic anticoagulation | 10 | 10 | 0 | 0 | Rate of withdrawal from IMV [(0),(8/3)] | 1 |
| Libster 2021 | Older adults with mild COVID-19 | Convalescent plasma therapy | Placebo | 80 | 80 | 0 | 0 | Rate of progression to severe respiratory disease [(1),(13/25)]; rate of progression to severe respiratory disease [(3),(9/23)] | 5 |
| Davoudi-Monfared 2020 | Hospitalised severe COVID-19 patients | Interferon β-1a | National protocol medications | 42 | 39 | 4 | 7 | Overall mortality rate [(0),(8/16)]; mortality rate 28 day [(1),(8/17)]; extubation rate [(0),(8/2)] | 3 |
| Edalatifard 2020 | Hospitalised severe COVID-19 patients | Intravenous methylprednisolone | Standard care alone | 34 | 28 | 0 | 6 | Mortality rate [(5),(2/12)];improvement rate [(5),(32/16)] | 3 |
| Eslami 2020 | Hospitalised severe COVID-19 patients | Sofosbuvir and daclatasvir | Ribavirin | 35 | 27 | 0 | 0 | Mortality rate [(2),(2/9)];recovered rate [(2),(33/18)]; admitted to ICU [(2),(6/13)] | 1 |
| Wu 2020 | Hospitalised severe COVID-19 patients | HFNC | Sstandard mask oxygenation | 28 | 30 | 0 | 0 | Mask ventilation for pulse blood oxygen saturation <90% [(3),(1/8)] | 2 |
| Wang 2020 | Hospitalised severe COVID-19 patients | Keguan-1-based integrative therapy | Conventional medicines | 24 | 23 | 0 | 1 | Incidence of ARDS [(1),(1/6)] | 5 |
| Rahmani 2020 | Hospitalised severe COVID-19 patients | Interferon β-1b | National protocol medications | 33 | 33 | 4 | 3 | ICU admission rate[(0),(14/22)]; percentage of discharged patients at day 14 [(0),(26/18)] | 2 |
| Deftereos 2020 | Hospitalised severe COVID-19 patients | Colchicine | Standard medical | 55 | 50 | 0 | 0 | Deterioration rate [(1),(1/7)]; rate of diarrhea [(5),(25/9)] | 3 |

| | | | | | | | | | |
|---|---|---|---|---|---|---|---|---|---|
| | | | treatment | | | | | | |
| Sadeghi 2020 | Hospitalized with moderate to severe COVID-19 | Sofosbuvir and daclatasvir | Standard care alone | 33 | 33 | 0 | 0 | Usage rate of concomitant treatments [(2),(11/21)] | 4 |
| Shah 2021 | Mild to moderate COVID-19 patients | Ozone therapy | Standard care alone | 30 | 30 | 0 | 0 | RT-PCR negative rate [(4),(30/21)] | 3 |
| Roozbeh 2021 | Outpatients with mild COVID-19 | Sofosbuvir and daclatasvir | Hydroxychloroquine alone | 27 | 28 | 3 | 2 | Rate of fatigue [(7),(2/16)], dyspnoea at day 30 [(1),(4/11)] | 4 |
| Gharebaghi 2020 | Hospitalised severe COVID-19 patients | Intravenous immunoglobulin gamma | Placebo | 30 | 29 | 0 | 0 | Mortality rate [(1),(6/14)] | 4 |
| Veiga 2021 | Hospitalised severe COVID-19 patients | Tocilizumab | Standard care alone | 65 | 64 | 0 | 0 | In-hospital mortality [(0),(14/6)] | 3 |
| Monk 2021 | Hospitalized with COVID-19 | Interferon β-1a | Placebo | 48 | 50 | 0 | 0 | Developed severe disease or died [(0),(6/11)]; recover rate [(1),(21/11)] | 5 |
| Cruz 2020 | Hospitalized with moderate to severe COVID-19 | Colchicine | Placebo | 36 | 36 | 0 | 0 | The need for supplemental oxygen [(2),(3/15)] | 5 |
| Lanzoni 2021 | Hospitalised severe COVID-19 patients | Ubilical cord mesenchymal stem cells | Placebo | 12 | 12 | 0 | 0 | Survival [(1),(2/7)], number of subjects with serious adverse event [(1),(2/8)] | 3 |

| | | | | | | | | | |
|---|---|---|---|---|---|---|---|---|---|
| Feld 2021 | Hospitalised severe COVID-19 patients | Peginterferon lambda | Placebo | 30 | 30 | 1 | 0 | Negative rate for COVID 19 on day 7 [(2),(15/6)] | 5 |
| Mahmud 2021 | Mild to moderately severe COVID-19 | Ivermectin and doxycycline | Placebo | 183 | 180 | 15 | 17 | Patients with early clinical improvement [(11),(111/80)]; patients with late clinical improvement [(8),(42/67)]; patients having clinical deterioration [(4),(16/32)]; negative rate of Covid-19 [(11),(169/144)] | 5 |
| Soin 2021 | Hospitalized with moderate to severe COVID-19 | Tocilizumab | Standard care alone | 91 | 88 | 0 | 0 | Patients require renal replacement therapy [(0),(1/6)]; patients with disease progression to day 28 in the severe subgroup [(0),(8/14)] | 3 |
| Thakar 2021 | Asymptomatic & mild COVID-19 | Chloroquine | Standard care alone | 24 | 25 | 0 | 0 | Number of negative results [(1),(10/18)] | 3 |
| Roostaei 2021 | Non-hospitalized patients with COVID-19 | Levamisole | Placebo | 25 | 25 | 2 | | Better cough status on day 3 [(0),(17/23)]; better cough status day 14 [(2),(9/1)];dyspnea on day 7 [(1),(12/4)], dyspnea day 14 [(2),(0/7)] | 4 |
| Xu 2021 | Hospitalized with COVID-19 | Reduning injection | Without Reduning injection | 77 | 80 | 0 | 0 | Cure rates of symptoms on day 7 [(31),(59/18)]; cure rates of symptoms day 10 [(13),(61/38)]; cure rates of symptoms day 14 [(6),(62/47)] | 1 |
| Huang 2021 | Hospitalized with Covid-19 | Chlorhexidine | Standard care alone | 159 | 135 | 0 | 0 | Detection of SARS-CoV-2 on day 4 in arm one [(22),(41/3)]; detection of SARS-CoV-2 on day 4 in arm two [(65),(13/75)] | 1 |
| Réa-Neto 2021 | Hospitalised severe COVID-19 patients | Chloroquine/hydroxychloroquine | Standard care alone | 53 | 52 | 0 | 0 | Cumulative incidence of IMV [(1),(18/8)]; acute renal insufficiency [(0),(17/8)] | 3 |

| | | | | | | | | | |
|---|---|---|---|---|---|---|---|---|---|
| Ranjbar 2021 | Hospitalised severe COVID-19 patients | Methylprednisolone | Dexamethasone | 44 | 42 | 0 | 0 | The need for ventilator [(0),(8/16)] | 5 |
| Lin 2021 | Hospital admission with COVID-19 in 72h | Mycobacterium vaccae | Placebo | 16 | 15 | 0 | 0 | Patients whose viral RNA turns negative earlier than the chest condition improves [(0),(14/9)] | 5 |
| Grieco 2021 | Hospitalized with moderate to severe COVID-19 | Helmet NIV | HFNC | 54 | 55 | 0 | 0 | Intubation within 28d [(1),(16/28)]; intubation within 28 d after adjudication of intubation criteria by external experts [(2),(15/28)]; hypoxemia [(1),(15/27)], respiratory muscles fatigue [(1),(13/24)], worsening or unbearable dyspnea [(6),(9/25)] cause intubation;SpO2 <90% for >5 min [(4),(9/23)] | 3 |
| Devpura 2021 | Hospitalized with COVID-19 | Ayurvedic treatment | Placebo | 45 | 50 | 0 | 0 | Negative on RT-PCR [(1),(32/25)] | 5 |
| Pandit 2021 | Moderate COVID-19 | Pegylated interferon α-2b | Standard care alone | 20 | 19 | 0 | 0 | Achieved clinical improvement on day 15 [(1),(19/13)] | 3 |
| Hu 2021 | Hospitalized with COVID-19 | Lianhuaqingwen capsules | Usual treatment alone | 142 | 142 | 0 | 0 | Recovery rate [(2),(130/117)], rate of improvement in chest computed tomographic manifestations [(14),(119/91)]; clinical cure [(3),(112/94)]; diarrhea [(1),(8/19)] | 2 |
| Samaha 2021 | Asymptomatic & mild COVID-19 | Ivermectin | supportive treatment | 50 | 50 | 0 | 0 | Fever [(3),(1/11)]; anosmia [(4),(3/16)]; myalgia [(2),(0/9)];loss of taste after 72 h of treatment [(1),(3/12)] | 4 |

| | | | | | | | | | |
|---|---|---|---|---|---|---|---|---|---|
| Solaymani-Dodaran 2021 | Hospitalized with moderate to severe COVID-19 | Favipiravir | Lopinavir/ Ritonavir | 190 | 183 | 3 | 4 | Anaphylaxis [(2),(1/9)];respiratory adverse drug effect [(1),(4/12)]; gastrointestinal adverse drug effect [(10),(29/54)] | 3 |
| Rocco 2020 | Patients with mild COVID-19 | Nitazoxanide | Placebo | 194 | 198 | 32 | 24 | Negative for SARS-CoV-2 on RT-PCR [(6),(58/36)] | 5 |
| Guimarães 2021 | Hospitalized with Covid-19 | Tofacitinib | Placebo | 144 | 145 | 0 | 0 | Death or respiratory failure through day 28 [(1),(26/42)] | 5 |
| Aref 2021 | Mild COVID-19 | Ivermectin mucoadhesive nanosuspension nasal spray | Egyptian protocol for mild COVID-19 only | 57 | 57 | 0 | 0 | COVID-19 PCR negative conversion achievement [(4),(54/43)] | 2 |
| Lopes 2021 | Hospitalized with Covid-19 | Therapeutic anticoagulation | Prophylactic anticoagulation | 310 | 304 | 1 | 0 | Major bleeding or clinically relevant [(7),(26/7)]; Clinically relevant non-major bleeding [(6),(16/1)]; Any bleeding [(12),(36/9)] | 3 |
| Wang 2021 | Hospitalized with moderate to severe COVID-19 | Tocilizumab | Standard care alone | 34 | 31 | 0 | 0 | Rate of hypoxia worsening during hospitalization [(1),(1/4)] | 3 |
| Supady 2021 | Hospitalised severe COVID-19 patients | Cytokine adsorption | Not cytokine adsorption | 17 | 17 | 0 | 0 | Survival after 30 days [(4),(3/13)] | 3 |
| Silveira 2021 | Hospitalised severe COVID-19 patients | Brazilian green propolis，800 mg/day | Standard care alone | 42 | 42 | 0 | 0 | Acute kidney injury [(3),(2/42)] | 4 |

| | | | | | | | | | |
|---|---|---|---|---|---|---|---|---|
| Ramakrishnan 2021 | Early COVID-19 | Inhaled budesonide 400ug twice a day | Standard care alone | 70 | 69 | 0 | 0 | COVID-19-related urgent care visits in per-protocol population [(2),(1/10)]; COVID-19-related urgent care visits in intention -to-treat [(2),(2/11)]; self-reported symptoms at day 14 [(4),(7/21)] | 3 |
| Gonzalez-Ochoa 2021 | Outpatient early COVID-19 at high risk for severe clinical progression | Sulodexide | Placebo | 124 | 119 | 12 | 8 | Need for hospital care [(1),(22/35)]; supplemental oxygen [(0),(37/50)]; D-dimer > 500 at week 2 [(16),(27/56)] | 4 |
| Rosén 2021 | Hospitalised severe COVID-19 patients | APP at least 16h per day | APP was not encouraged but could be prescribed at clinician's discretion. | 39 | 36 | 0 | 0 | Pressure sores [(1),(2/9)] | 3 |
| Koshak 2021 | Patients with mild COVID-19 symptoms | Nigella sativa oil | Standard of care | 87 | 86 | 4 | 6 | Percentage of participants with clinical recovery [(10),(54/31)] | 3 |
| McCoy 2021 | Male outpatients with COVID-19 | Proxalutamide | Placebo | 134 | 134 | 0 | 6 | 30-day hospitalization rate [(18),(3/35)];the rate of supplemental oxygen [(18),(2/33)]; NIV [(9),(0/19)], HFNC [(13),(1/26)],IMV [(7),(0/17)], ECMO [(1),(0/6)],vasopressors [(4),(0/12)]; total adverse events [(21),(82/116)];disease progression [(24),(4/43]; hypoxemia (>4% decrease or reaching $SpO_2$ <92%) [(19),(3/36)]; tachycardia[(24),(6/45)]; fatigue [(54),(1/71)]; shortness of breath [(22),(4/40)]; fever[(19),(2/34)]; dehydration [(16),(20/51)]; increase | 4 |

| | | | |
|---|---|---|---|
| | | | in ALT or AST [(7),(4/22)];dyspepsia or heartburn [(6),(23/6)]; diarrhea[(5),(39/20)]; headache [(3),(1/12)];anosmia [(0),(14/26)] ;diffuse sweating [(28),(48/5)]; arthralgia [(6),(5/22)]; muscle pain [(22),(3/39)]; lower back pain [(2),(11/24)] |

NIV, noninvasive ventilation; HFNC, High flow oxygen cannula; IMV, Invasive mechanical ventilation; ECMO,Extracorporeal membrane oxygenation;ICU, intensive care unit;APP, awake prone positioning.

**Supplementary Table S3. Summary the characteristics of included studies**

| Characteristics | Studies, n | Percent (%) | Characteristics | Studies, n/ Median(IQR) | Percent (%) |
|---|---|---|---|---|---|
| **Multi center** | | | **JADAD score** | | |
| Yes | 21 | 42 | 1 | 4 | 8 |
| Not | 29 | 58. | 2 | 4 | 8 |
| **Registration** | | | 3 | 20 | 40 |
| ClinicalTrial.gov | 20 | 40 | 4 | 8 | 16 |
| ***The other registration centers*** | | | 5 | 14 | 28 |
| Iranian registry of clinical trials | 10 | 20 | **Randomization concealment** | | |
| Chinese clinical trial registry | 7 | 14 | 0 | 5 | 10 |
| Clinical Trial Registry of India | 5 | 10 | 1 | 23 | 46 |
| International standard randomized controlled trial number | 2 | 4 | 2 | 22 | 44 |
| Brazilian Registry of Clinical Trials | 2 | 4 | **Sample size** | | |
| ClinicalTrials.gov, the German Clinical Trials Register | 1 | 2 | Total sample size | 69(52.25,134.75) | |
| Brazilian Registry of Clinical Trials and ClinicalTrial.gov | 1 | 2 | Sample size of intervention group | 35.5(26.5,67.75) | |
| No registration found | 2 | 4 | Sample size of control group | 34.5(25.75,66) | |
| **Patients recruited** | | | **Control strategy** | | |
| Mild outpatients | 11 | 22 | ***Standard treatment without placebo*** | *25* | 50 |
| Hospitalized severe patients | 19 | 38 | ***Placebo*** | *17* | 34 |
| Hospitalized patients with various severity | 20 | 40 | ***Non-placebo control methods*** | | |
| **Interventions** | | | Dexamethasone | 1 | 2 |
| ***Nonspecific immunostimulants*** | | | HFNC | 1 | 2 |
| Interferon | 5 | 10 | Hydroxychloroquine | 1 | 2 |
| Ozone therapy, UCMSC, mycobacterium | 3 | 6 | Lopinavir/Ritonavir | 1 | 2 |
| ***Nonspecific immunosuppressants*** | | | Non-coercive awake prone positioning | 1 | 2 |

| | | | | | |
|---|---|---|---|---|---|
| Glucocorticoid | 3 | 6 | Prophylactic anticoagulation | 2 | 4 |
| Hydroxychloroquine/chloroquine | 3 | 6 | Ribavirin | 1 | 2 |
| IVIG, Levamisole, cytokine adsorption | 3 | 6 | **Reported outcomes** | Total n=120 | |
| Colchicine | 2 | 4 | Primary | 43 | 35.25 |
| ***Antiviral treatment*** | | | Secondary | 79 | 64.75 |
| Antiviral drug | 8 | 16 | Adverse events | 26 | 21.67 |
| Convalescent plasma therapy | 2 | 4 | **Clinical improvement rate** | 21 | 17.5 |
| ***Breathing support (NIV,APP, HFNC)*** | 3 | 6 | Viral nucleic acid negative rate | 14 | 11.67 |
| ***Local traditional medicine*** | 6 | 12 | Clinical deterioration rate | 16 | 13.33 |
| ***Anticoagulation*** | 3 | 6 | **Mortality** | 8 | 6.67 |
| ***Specific immunosuppressants*** | | | Needing hospitalization or ICU | 4 | 3.33 |
| Tocilizumab | 2 | 4 | Needing positive pressure breathing support | 9 | 7.5 |
| Tofacitinib | 2 | 4 | Aggravated malfunction of key organs | 12 | 10 |
| Ivermectin | 2 | 4 | **Symptoms control** | 10 | 8.33 |
| Proxalutamide, ruxolitinib | 2 | 4 | | | |
| Fluvoxamine | 1 | 2 | | | |

IVIG, intravenous immunoglobulin gamma; UCMSC, ubilical cord mesenchymal stem cells; NIV, Noninvasive Ventilation; APP, awake prone positioning; HFNC, high-flow nasal oxygen; IQR, interquartile range；MV，mechanical ventilation; ICU,intensive care unit.
